# Bictegravir Plus Tenofovir Alafenamide Nanoformulation as a Long-Acting Pre-Exposure Prophylaxis Regimen: Application of Modeling to Design Non-Human Primate Pharmacokinetic Experiments

**DOI:** 10.3389/fphar.2020.603242

**Published:** 2020-12-18

**Authors:** Simone Perazzolo, Subhra Mandal, Pavan K. Prathipati, Christopher J. Destache

**Affiliations:** ^1^ Nanomath LLC and University of Washington, Seattle, WA, United States; ^2^ School of Pharmacy, Creighton University, Omaha, NE, United States

**Keywords:** pre-exposure prophylaxis, HIV, mechanism-based pharmacokinetic modeling, bictegravir, tenofovir alafenamide

## Abstract

Bictegravir (BIC) and tenofovir alafenamide fumarate (TAF), two potent anti-HIV drugs, had been nanoformulated (*nBIC-TAF*) to achieve once-a-month PrEP coverage. *In-vivo* mouse experiments for *nBIC-TAF* exhibited favorable subcutaneous (SC) pharmacokinetics. To probe the clinical suitability of the *nBIC-TAF*, as the next step, we intend to study *nBIC-TAF* in non-human primates (NHP), as the best preclinical model to foster clinical trials. Before entering an expensive NHP study, however, we seek to improve our a priori understanding about *nBIC-TAF* in higher species, having just mouse data. The mechanism-based pharmacokinetic modeling (MBPK) has been used as an appropriate method for pharmacokinetic modeling and interspecies scaling for nanoformulations. Via the use of MBPK, in this work, we created a model for *nBIC-TAF* able to predict plasma concentration-time curves in NHP. BIKTARVY is a daily oral combination of BIC, TAF, and emtricitabine (Gilead Science, CA), approved for HIV therapy. Using BIKTARVY equivalent dosages (from their NHP studies), we predicted that, following just one SC dose of *nBIC-TAF* in NHP, both BIC and tenofovir will have detectable and above *in vitro* efficacy levels for 28 days. Furthermore, the MBPK was able to provide a mechanistic explanation regarding the long-acting mechanism characterizing *nBIC-TAF*: nanoparticles stores in the SC space from which drugs slowly dissociate. Dissociated drugs in the SC space then buffer the plasma pool over time, yielding an extended-release effect in the plasma. Overall, we predicted for *nBIC-TAF* a promising long-acting pharmacokinetic in NHP, potentially usable as monthly PrEP. These results will help investigators to gain confidence for facing regulatory submissions at early stages.

## Introduction

Effective prevention strategies for HIV are a critical public health priority worldwide, including the United States (Chou et al., 2019). In the absence of an effective HIV cure, research into potential preventative strategies is critical. The long-acting antiretroviral therapy (LA ART) proposes the formulation of antiretrovirals into nanoparticles to prolong their pharmacokinetic and antiretroviral activity while improving patient adherence. Of interest, it is the use of long-acting strategies as a preventative modality for pre-exposure prophylaxis (PrEP). Previous PrEP trials have documented the importance of adherence for adequate protection and a very positive acceptance of LA ART solutions among people living with HIV (Fonner et al., 2016). In the ÉCLAIR study, the majority of participants that received an LA ART intramuscular injections of cabotegravir 800 mg every 12 weeks were satisfied and willing to continue or would recommend the therapy (Murray et al., 2018). Long-acting cabotegravir as a single agent for PrEP may be effective for both men and cisgender women, but it might also have some pharmacokinetic characteristics that researchers do not fully understand (Scarsi, 2020).

Our research into different nanoparticle combinations for PrEP has led to a novel nanoformulation that combines bictegravir (BIC) with tenofovir alafenamide fumarate (TAF), referred here as nanoformulated-BIC-TAF, or *nBIC-TAF* (Mandal et al., 2019; Mandal et al., 2020). *nBIC-TAF* may be a suitable combination for PrEP. BIC is a novel and very potent integrase strand transfer inhibitor (Smith et al., 2018). TAF is a tenofovir (TFV) prodrug; TFV is a well-consolidated nucleotide reverse transcriptase inhibitor. TAF is of interest to the HIV community for PrEP since it has been FDA-approved for men who have sex with men and transgender women.

Several different platforms are being investigated for TAF delivery for PrEP including polymeric drug delivery implants, nanofluidic implants, as well as osmotic pumps (Gunawardana et al., 2015; Schlesinger et al., 2016; Johnson et al., 2019; Chua, et al., 2018). Each of these platforms have advantages and disadvantages, offering different technologies to produce sustained-release for tenofovir and the active metabolite tenofovir diphosphate. However, this report describes the use of TAF + BIC combination for PrEP as a sustained subcutaneous (SC) delivery system. This is the first report of using combination of two different classes of HIV drugs formulated as a sustained-release nanoformulation for PrEP.

The use of *nBIC-TAF* extended-release nanoformulation will be for SC administration. SC injections may be more user-friendly to allow the PrEP individual to control their administrations. *Additionally, SC administration of nanoparticles may result in more favorable drug pharmacokinetics, particularly, longer release time and better lymphatic targeting* (Perazzolo et al., 2018; Perazzolo et al., 2020). *nBIC-TAF* nanoparticles are made of poly(lactic-co-glycolic acid, PLGA) as the main polymer in which BIC and TAF are co-formulated (Mandal et al., 2020). PLGA is an FDA-approved polymer owing to its biodegradability and biocompatibility. Using the oil-in-water emulsion technique and lyophilization, the *nBIC-TAF* formulation is thus produced (Mandal et al., 2020).

We have reported *nBIC-TAF in vivo* pharmacokinetics and biodistribution in mice (Mandal et al., 2020). In mice, *nBIC-TAF* demonstrated long-acting features including sustaining plasma levels of BIC and TFV for 1 month after injection (BIC:TAF, 1:1 M ratio, dose = 200 mg/kg, ∼ 1 mg) (Mandal et al., 2020). In bringing forward *nBIC-TAF* to non-human primates (NHP) testing, as the best animal model for HIV therapy/PrEP preclinical assessments, many unknowns surround the choice of the NHP regimen. Nanoformulated drugs have a high degree of pharmacokinetic unpredictability when scaled from smaller to higher species, and therefore it can be inappropriate to use standard interspecies scaling rules for nanoformulated drugs (Valic and Zheng, 2019). Additionally, NHP experiments are costly and beg ethical concerns, thus dose-escalation assessments in NHP could become burdensome.

In the absence of an empirical method to scale pharmacokinetic parameters from mice to NHP for nanoformulated drugs, the computational mechanism-based pharmacokinetic modeling (MBPK) may be the ideal asset (Perazzolo et al., 2018; Perazzolo et al., 2020). The MBPK modeling consists of the integration of classical pharmacokinetic modeling with elements based on the main mechanisms controlling the drug prolonged pharmacokinetics. For example, *Perazzolo et al.* investigated the targeted long-acting features of their lipid-based nanoparticle formulation in NHP. Using the MBPK modeling, they explained that their nanoparticles were taken up in the lymphatic system where they formed a pre-systemic depot controlling the observed prolonged plasma pharmacokinetics in NHP (Perazzolo et al., 2020).

In this work, we explore the potential of *nBIC-TAF* to achieve a pharmacokinetic suitable for a 1-month PrEP coverage in NHP. NHP projections not only will guide the design of NHP trials, but it can be valuable to hypothesize how *nBIC-TAF* performs in humans at this very early stage.

## Methods

### nBIC-TAF Formulation

BIC and TAF loaded nanoformulation were fabricated using previously reported standardized nanoformulation methods (Mandal et al., 2019; Mandal et al., 2020).

### Setting Up the Mechanism-Based Pharmacokinetic Model for nBIC-TAF

The a priori plasma-concentration predictions in NHP were carried out by the predictive pharmacokinetic computational modeling (viz. mechanism-based pharmacokinetics, or MBPK (Perazzolo et al., 2020). MBPK is the combination of classical pharmacokinetic modeling with elements describing the main mechanisms working in the system. MBPK founds on the mass conservation law and it has been used to inform several biological systems (Lofthouse et al., 2015; Perazzolo et al., 2015; Perazzolo et al., 2017; Perazzolo, 2017; Gázquez et al., 2019). Modeling procedure consisted of four steps: 1) Model structure building to represent the absorption mechanisms able to explain the long-acting pharmacokinetics exhibited by the nanoformulation; 2) Identifying the model parameters with mouse data; 3) Scaling the mouse model to get an NHP model; 4) Simulate a reference dose in the NHP model and observe results. The modeling effort was carried out using SAAM II (The Epsilon Group, VA). Because BIC-TAF drug-drug interaction should not be expected (Gilead Sciences, 2012), we modeled the two drugs as pharmacokinetically independent. Because TAF is the tenofovir prodrug (TFV), a sub-model for TFV was also included. Linear kinetics was assumed.

### Defining Mechanism-Based Pharmacokinetic Model Structure

The modeling schematics are depicted in Supplementary (Supplementary Figures S1,S2). BIC from *nBIC-TAF* was modeled by three compartments: two SC compartments in the Injection Side of the model (Supplementary Figure S1, left-hand side), and a one-compartment system on the Systemic Side (Supplementary Figure S1, right-hand side). The *nBIC-TAF* dose is imputed in the NP-associated BIC compartment (*nBIC*_*sc*_) from which BIC dissociates locally to the other SC compartment for free BIC (*BIC*_*sc*_). From *BIC*_*sc*_, BIC then moves to the central pool via a linear absorption rate. From the one-compartment system in the Systemic Side (*BIC*_*c*_), BIC eliminates. According to the *in vitro* dialysis experiment for stability efficiency (i.e., the degree of association in formulation), BIC has an entrapment efficiency as EE = 58% with the nanoparticles (Mandal et al., 2020). Hence, we divided the BIC bolus dose in half between *nBIC*_*sc*_ and *BIC*_*sc*_ compartments as initial conditions.

TAF was modeled by four compartments (Supplementary Figure S2). Three SC compartments on the Injection Side (Supplementary Figure S2, left-hand side) and a one-compartment system on the Systemic Side (Supplementary Figure S2, right-hand side). The *nBIC-TAF* dose is imputed in the NP-associated TAF compartment (*nTAF*_*sc*_) from which TAF dissociates locally to the other TAF SC compartment for free TAF (*TAF*_*sc*_). From *TAF*_*sc*_, TAF then either moves to the TAF central pool (*TAF*_*c*_) via a linear absorption rate or converts locally to TFV, represented as an SC TFV compartment (*TFV*_*sc*_ *)*. The TAF- > TFV conversion rate constant is denoted by the k_reac_. TAF on the Systemic Side, *TAF*_*c*_, can either eliminate via its one-compartment system or covert into TFV. *TFV*_*sc*_ can then be taken up in the Systemic Side into a TFV compartment (*TFV*_*c*_) where it can eliminate via a one-compartment system. Note that both TAF and TFV on the Injection Side required of a local distribution compartment to account for a biphasic decay exhibited by the injection site measurements (Supplementary Figure S3). According to the *in vitro* dialysis experiment for stability efficiency, TAF has an entrapment efficiency EE = 55%, therefore the input bolus dose was fractionated accordingly between *nTAF*_*sc*_ and *TAF*_*sc*_ compartments.

### Parameter Estimation

MBPK structural parameters were regressed from the geometrical mean of the timepoints from a previous mouse pharmacokinetic experiment where *nBIC-TAF* was given as a single-dose SC injection at 200 mg/kg (*∼*1 mg) for each BIC and TAF (Mandal et al., 2020). Hence, we got the *nBIC-TAF* MBPK model for a typical mouse of 23 mg body weight (BW). Additionally, to pin down TAF- > TFV conversion, some TAF-TFV parameters had been regressed from another dataset about TAF long-acting study in mice carried out by the same group (Prathipati et al., 2017).

### Scaling Up to Non-Human Primate

Having estimated a set of parameters identifying the mouse MBPK model we are now ready to scale this model up to predict the PK of *nBIC-TAF* in NHP. We scaled the mouse model to a typical NHP of 5 kg. The scaling procedure was taken from Biliouris et al. (2018). According to their rationale, volumes and eliminations were assumed to change accordingly to the animal BW. The model structure does not change between species nor does the absorption kinetics and subsequently the long-acting mechanism. Allometric scaling was based on the rule of exponents employed for volumes (one as the exponent), and the elimination rates from the central pool (0.85 as the exponent):

$$V_{c,NHP}=\;V_{c,mouse}{\left(\frac{5.0\;kg}{0.0175\;kg}\right)}^{1}\;$$ (1)

$$V_{sc,NHP}=\;V_{sc,mouse}{\left(\frac{5.0\;kg}{0.0175\;kg}\right)}^{1}$$ (2)

$$CL_{NHP}=\;K_{e,\;mouse}V_{c,mouse}{\left(\frac{5.0\;kg}{0.0175\;kg}\right)}^{0.85}$$ (3a)

$$K_{e,NHP}=\frac{CL_{NHP}}{V_{c,NHP}}$$ (3b)

with V_c_ and V_sc_ as the volumes on the Systemic Side and Injection Side, respectively. CL as the central compartment clearance as the product of V_c_ and the elimination from the central compartment rate K_e._ Second subscripts denote the animal (e.g., NHP: non-human primate).

As our first dose candidate, we chose to simulate, in the MBPK for NHP, an SC dose of 30 mg/kg for BIC, and 50 mg/kg for TAF. These dosages were taken from the BIKTARVY’s preclinical report in NHP (Gilead Sciences, 2012). BIKTARVY (Gilead, CA) is an FDA-approved triple-drug combination of BIC, TAF, and emtricitabine, all in one pill intended for a once-a-day HIV oral therapy regimen. Gilead carried out NHP experiments at these dosages and reported no adverse events. Hence, it could be considered a safe starting point for *nBIC-TAF*.

The comparison between *nBIC-TAF* (MBPK-simulated) and BIKTARVY (NHP trials) was assessed by non-compartmental analysis (NCA). NCA consists of a set of plasma-derived computations: maximal concentration at the peak, C_max_; the last point recorded T_end_, last concentration recorded C_max_, area under the curve (AUC_inf_) extrapolated to infinity; terminal half-life T_12,z_. NCA computations from MBPK-simulated plasma curves were done in Phoenix WinNonLin (Certara, NJ).

## Results

### nBIC-TAF Long-Acting Mechanism

**FIGURE 1:**
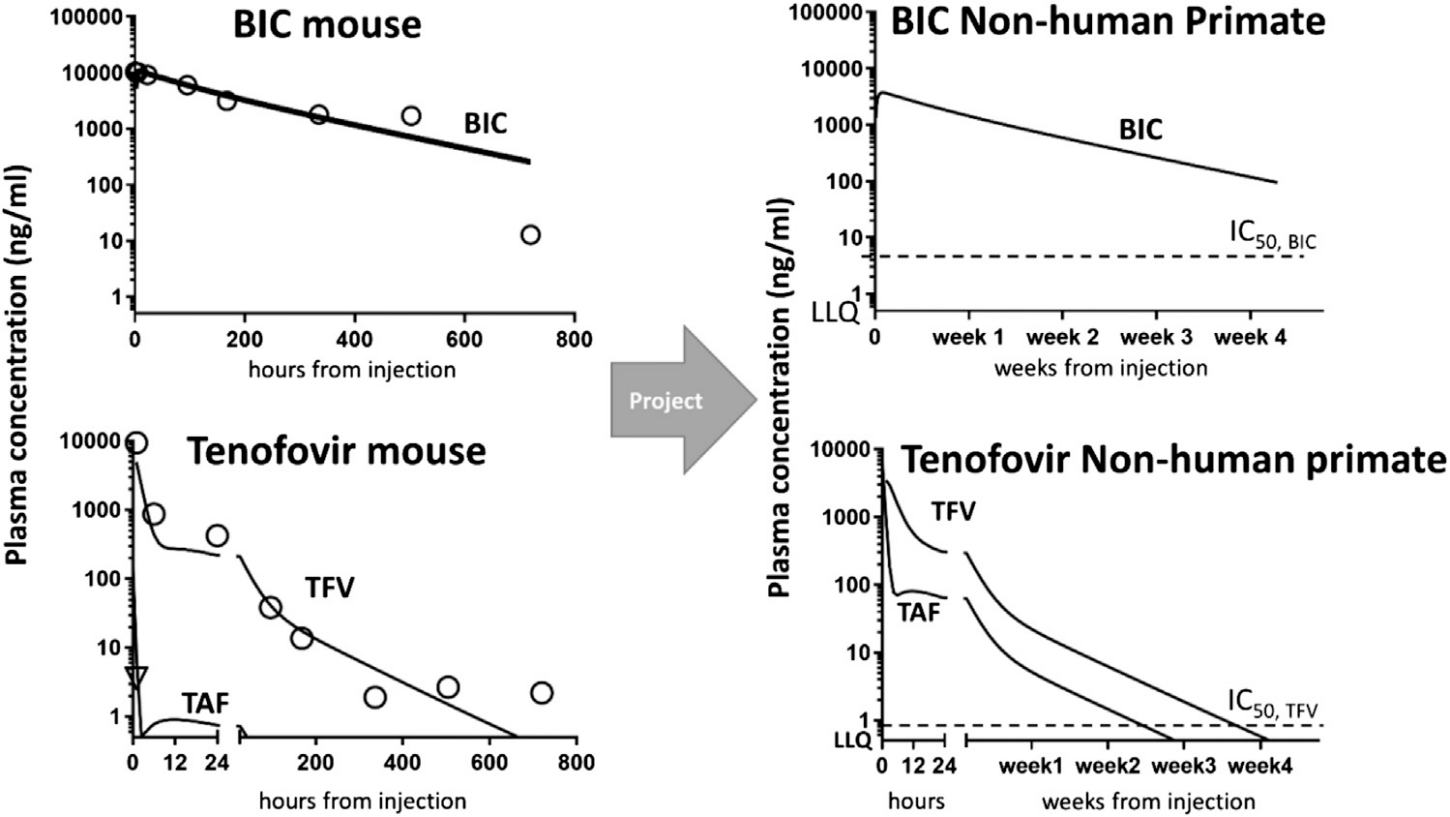
Non-human primate projections of concentration-time courses in the plasma from mouse data collection and modeling. **Left panel:** mouse data (symbols) vs. MBPK fit (solid lines). Model fitting of mouse data enabled the structure identification of the MBPK model for all drugs, i.e., BIC **(upper)** and TAF/TFV **(bottom)**. Right panel: MBPK-based concentration-time predictions in NHP at BIKTARVY’s equivalent doses: BIC **(upper)** and TAF/TFV **(bottom)**. Plotted also in-house *in vitro* IC50 for BIC and TFV (dashes) and Lower Limits of Quantification (LLQ = 0.5 ng/ml) overlapping with the *x*-axis. Predictions for a typical monkey of 5 kg.

The advantage of using the MBPK is that it can provide a mechanistic explanation about the long persistence of all drugs in the plasma exhibited by *nBIC-TAF*. The fit looked good for the plasma for all drugs (r2>0.89, Figure 1, left panel). The plasma fit was implemented at the same time with the injection site data fitting. Data fitting for the injection site resulted in good quality as well (r2 > 0.93, Supplementary Figure S3). The mechanism hypothesis testing was guided by the goodness of fit. The best structural model able to fit both the plasma and injection site data was the following: there is a nanoparticle depot adjacent to the site of injection that dissociates its payload to a local free pool; the local free pool buffers the plasma via the absorption of drugs to the central blood pool. Also, TAF and TFV both need a local distribution pool to characterize the typical biphasic pharmacokinetics of TFV (schemes depicted in Supplementary Figures S1, S2). Whereas other modeling configurations were attempted–maintaining modeling identifiability–the fit worsened and they could not explain simultaneously the plasma and injection site data time-courses. It is reasonable to assume, at this stage, that the long-acting working principle of *nBIC-TAF* conserves in higher mammals, such as NHP and perhaps humans. Model parameters estimates are reported with precision in Supplementary Table S1.

### nBIC-TAF Projections in Non-Human Primates

Simulating via MBPK a single-dose SC administration of *nBIC-TAF* at equivalent BIKTARVY’s dose in NHP, we predicted both BIC and TFV levels as reasonably sustaining concentrations up to 28 days (4 weeks). NHP projections in plasma indicated concentration above *in vitro* efficacy for both drugs (>IC_50_ = 4.3 ng/ml for BIC and 0.08 ng/ml for TFV, IC_50_ values from (Mandal et al., 2020), and detectability in plasma samples (>LLQ = 0.5 ng/ml). NHP projections are plotted in Figure 1, right panel.

The full time-course predictions in NHP can be used to carry out the non-compartmental analysis in NHP (NCA). The comparison of NCA parameters between single-dose *nBIC-TAF* and single-dose BIKTARVY is reported in Table 1. From *nBIC-TAF*, BIC’s C_max_ was lower than BIKTARVY’s C_max_ (3.7 vs. 19 μg/ml); The end concentrations C_end_ (i.e., concentrations at T_end_) were lower for BIC than BIKTARVY (0.94 vs. 2.3 μg/ml); Note, however, that we report C_end_ for BIC from *nBIC-TAF* at 28 days, whereas BIKTARVY’s C_end_ is at 1 day. Hence, the exposure of BIC from *nBIC-TAF* - calculated by simulated plasma AUC (to infinity) - was 6-folds higher than BIKTARVY’s BIC exposure, at the same dose. The NHP half-life of BIC in BIKTARVY is 3.3 h, whereas in our *nBIC-TAF* projections it was 330 h (100-fold expected enhancement).

**TABLE 1:** Comparison of BIC and TAF/TFV NCA parameters between MBPK-predicted *nBIC-TAF* and BIKTARVY in Non-human Primates.

Non-human primate NCA		
Formulation	Creighton U.’s ***nBIC-TAF* (MBPK-simulated)**	Gilead’s**BIKTARVY (label)**
Drug	BIC	BIC^a^
Dosage	30 mg/kg SC single-dose	30 mg/kg Oral single-dose
C_max_ (μg/ml)	3.7	19
T_end_ (h)	672	24
C_end_ (μg/ml)	0.94	2.3
AUC_inf_ (μg h/ml)	670	170
T_12_ (h)	330	3.3
Drug	TAF	TAF
Dosage	50 mg/kg SC single dose	50 mg/kg Oral single dose
C_max_ (μg/ml)	3.5	4.1
T_end_ (h)	672	-
C_end_ (μg/ml)	0.08	-
AUC_inf_ (μg h/ml)	15	3.9
T_12,z_ (h)	220	0.40
Drug	TFV	TFV
Cmax (μg/ml)	3.5	1.3
T_end_ (h)	672	-
C_end_ (μg/ml)	0.41^b^	-
AUC_inf_ (μg h/ml)	40	10
T_12,z_ (h)	226	17

LLQ = 0.50 ng/ml.

a BIC (Sodium Salt) from male cynomolgus monkeys following single oral doses in aqueous suspension. ^b^BIC (bictegravir, sodium salt) from male cynomolgus monkeys following single oral doses in aqueous suspension (Gilead Sciences, 2012; Perrazolo, et al., 2018).

Following a single-dose SC simulation of *nBIC-TAF*, we predicted TAF C_max_ slightly lower than BIKTARVY (3.5 vs. 4.1 μg/ml). Because TAF is a TFV prodrug, TFV pharmacokinetics was measured, modeled, and therefore extrapolated to NHP as well. Following a single-dose SC simulation of *nBIC-TAF*, TFV had approximately 2.5-folds higher C_max_ than BIKTARVY (3.5 vs 1.3 μg/ml). The C_end_ are not reported for BIKTARVY, nevertheless, most of TAF and TFV in BIKTARVY should be cleared by 24–48 h (Gilead Sciences, 2012; Perazzolo et al., 2018). At 28 days, MBPK projections for TFV were slightly over the LLQ = 0.50 ng/ml (i.e. C_end_ = 0.53 ng/ml). We hit the LLQ limit at 690 h (∼29 days), which is close enough to the month extended-release effect. Hence, the exposure of TFV from *nBIC-TAF* - calculated by simulated plasma AUC (to infinity) - was 4-fold higher than BIKTARVY. The NHP half-life of TAF and TFV in BIKTARVY are 0.40 and 17 h, respectively, whereas in our *nBIC-TAF* projections TAF and TFV, T_12_ was 220 and 226 h, respectively (>100-fold and ∼13-fold expected enhancements).

Overall, *nBIC-TAF* predictions in NHP - based on mouse data - project sustained release in terms of AUC and T_12_ for both drugs. With the scope of designing a 1-month PrEP coverage trial for NHP, 30 mg/kg BIC in *nBIC-TAF* could be a reasonable dose; 50 mg/kg for TAF in *nBIC-TAF* could be a reasonable lower-boundary dose for TFV. According to Gilead (Gilead Sciences, 2012), both dosages are safe in NHP.

## Discussion

Based on mouse pharmacokinetic data, typically available in the lab, we have informed the investigators how their (nano) product may behave and work NHP to achieve 1-month HIV PrEP. Having this valuable information, at these early development stages, will improve developer confidence in regulatory acceptance.

At the equivalent of one dose of BIKTARVY, *nBIC-TAF* was predicted to have sustained and likely effective levels for 4 weeks in NHP. If confirmed by successive NHP studies, the potential of using one *nBIC-TAF* dose in place of 30 BIKTARVY pills for PrEP can, therefore, be hypothesized in humans. However, some caveats should be expected when extrapolating from NHP to humans. BIC metabolism or TFV active transport activities may change, being lower and higher respectively. Once NHP data will be collected, these physiological differences in primate-to-human scaling can be explored and accounted for in a model-based scaling procedure by integrating the current MBPK knowledge with physiologically-based pharmacokinetic modeling (PBPK).

Several previous studies have documented additive toxicity when TAF was injected SQ. The authors have *in vitro* evidence that the polymer reduces the cytotoxicity in epithelial cells from TAF (Mandal et al., 2020). Additionally, photos were taken at the SQ injection site at the time of euthanasia for the mice that underwent the PK studies. There were no evidence of any toxicity at the injection site over the 30-days trial (Mandal et al., 2020).

Furthermore, the active intracellularly phosphorylated ester of TFV, the TFV-diphosphate (TFVdp), should also be included in the discussion. TFVdp in peripheral blood mononuclear cells is reported as 30–60% of the parent drug in the plasma of NHP (Anderson et al., 2011). Because the working mechanism of *nBIC-TAF* entails that TFV in plasma is the free form (i.e., not bound to particles), plus the fact that TFV is scarcely bound with serum proteins, we can speculate that TFVdp levels from an *nBIC-TAF* administration in target mononuclear cells might be also sustained at a reasonable level for the prolonged period of coverage.

Overall, we forecast using computational pharmacokinetic modeling, MBPK, the NHP time-concentrations of a novel *nBIC-TAF* nanoformulation. The potential predicted for *nBIC-TAF* is for 1-month coverage after one single injection at the same dose of an FDA-approved drug orally given daily. Awaiting NHP primate pharmacokinetic results we provided herein a priori guidance on the first-in-monkey dose, as well as a biological working mechanism to explain the long-acting pharmacokinetics of *nBIC-TAF,* both valuable assets in strategic and lean drug development.

## Data Availability Statement

The raw data supporting the conclusions of this article will be made available by the authors, without undue reservation.

## Ethics Statement

The animal study was reviewed and approved by Creighton University Animal Research Committee (protocol #1111).

## Author Contributions

SM and PP performed experiments and analyzed data; edited manuscript; SP performed analysis and modeled data and wrote manuscript; CD performed experiments and edited manuscript.

## Conflict of Interest

SP is employed by the company NanoMath, LLC.

The remaining authors declare that the research was conducted in the absence of any commercial or financial relationships that could be construed as a potential conflict of interest.

## Abbreviations

AUC, area under the curve (to infinity); BIC, bictegravir; LA ART, long-acting antiretroviral therapy; MBPK, mechanism-based pharmacokinetic (model); NHP, non-human primate; nBIC-TAF, nanoformulated-BIC + TAF; PrEP, pre-exposure prophylaxis; T12, half-life; TAF, tenofovir alafenamide fumarate; TFV, tenofovir.
