# Supplementary material for: Bictegravir Plus Tenofovir Alafenamide Nanoformulation as a Long-Acting Pre-Exposure Prophylaxis Regimen: Application of Modeling to Design Non-Human Primate Pharmacokinetic Experiments
Source: Front Pharmacol. 2020 Dec 18;11:603242. doi: 10.3389/fphar.2020.603242 (PMC7775496; doi:10.3389/fphar.2020.603242)
Supplement: Supplementary file 1 [file datasheet1.docx]

Supplementary Material

# Supplementary Data

Supplementary Table 1*. PK modeling estimate results and monkey scaling of scalable parameters as per Equations 1-3 in the main text*

| **Mice** (BW=17.5g) | TAF | | TFV | BIC |
| --- | --- | --- | --- | --- |
| Vc (ml) | 1.0e3 ± 1.0e2^a^ | | 1.2 ± 0.9 | 3.0 ± 1.0 |
| Vsc (ml) | 68 ± 18 | | 0.001 ± 1e-4 | 9.8 ± 0.8 |
| K_abs_ (h^-1^) | 0.14 ± 0.06 | | 0.044 ± 0.009 | 0.012 ± 0.004 |
| K_diss_ (h^-1^) | 0.029 ± 0.008 | | - | 0.0046 ± 0.006 |
| K_reac1_ (h^-1^) | 6.6e-4 ± 2.0e-4 | | - | - |
| K_reac2_ (h^-1^) | 0.012 ^b^ | | - | - |
| K_e_ (h^-1^) | 3.0 ^b^ | | 0.62 ^b^ | 0.61 ± 0.15 |
| **Monkey** (BW=5kg) | | TAF | TFV | BIC |
| Vc (ml) | 28e4 | | 342 | 857 |
| Vsc (ml) | 1.9e4 | | 0.28 | 2800 |
| K_e_ (h^-1^) | 1.3 | | 0.27 | 0.27 |

Estimaties with estimation precision (SD). Measurement error as standard CV=10% in SAAMII.

**a** TAF central volume results much higher than mouse plasma volume (~1.5ml). Note that TAF Vc estimate was driven by the fact that it dropped immediately to close to undetectablilty limits between 1 and 6 hours from SC injection. Additionally, TAF may have V of distribution trending high(Markowitz et al., 2014).

**b** Values estimated from (Prathipati et al., 2017).

## Supplementary Figures


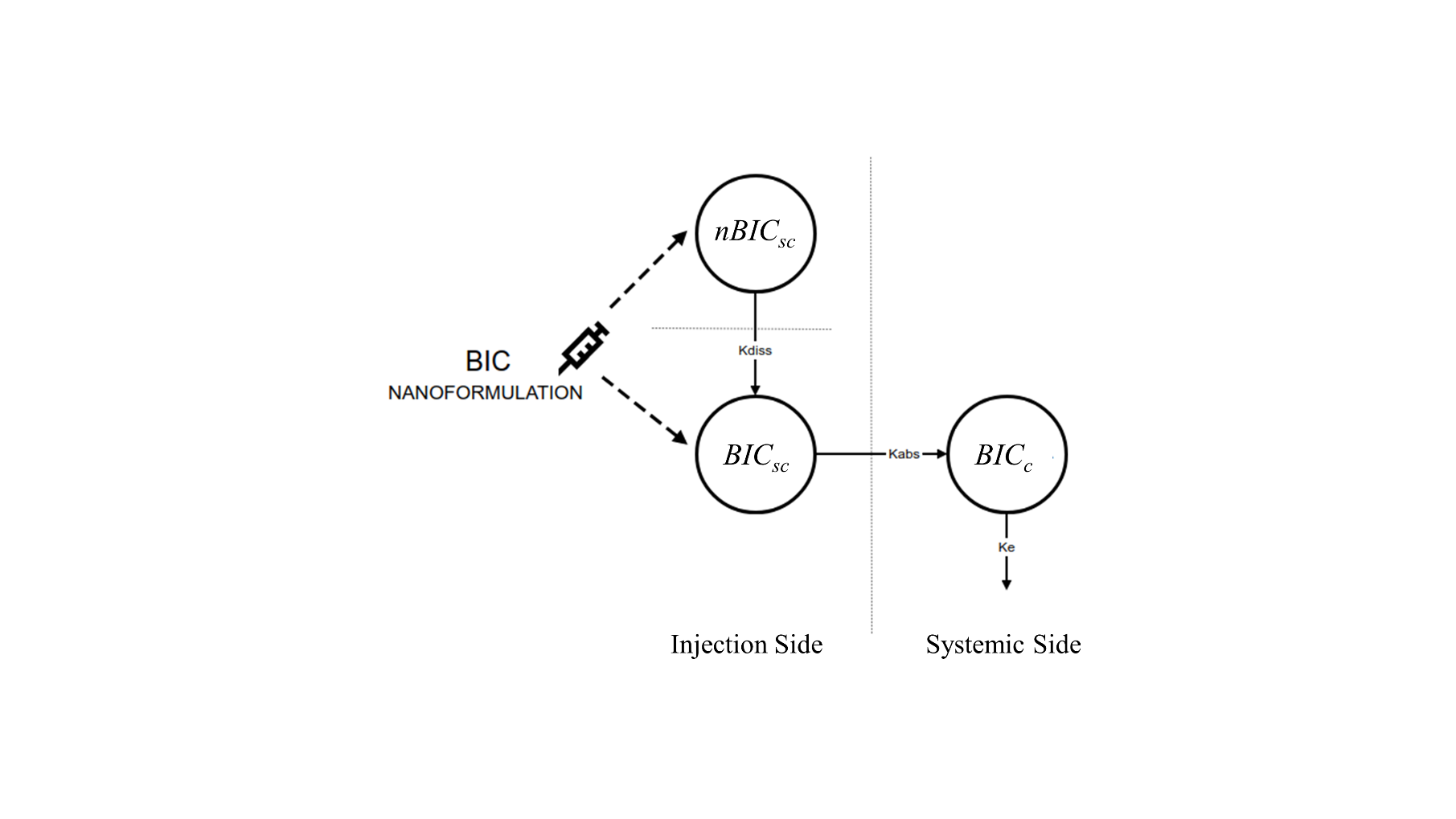


*Supplementary Figure 1.* MBPK schematics for nanoformulated BIC in the nBIC-TAF formulation.


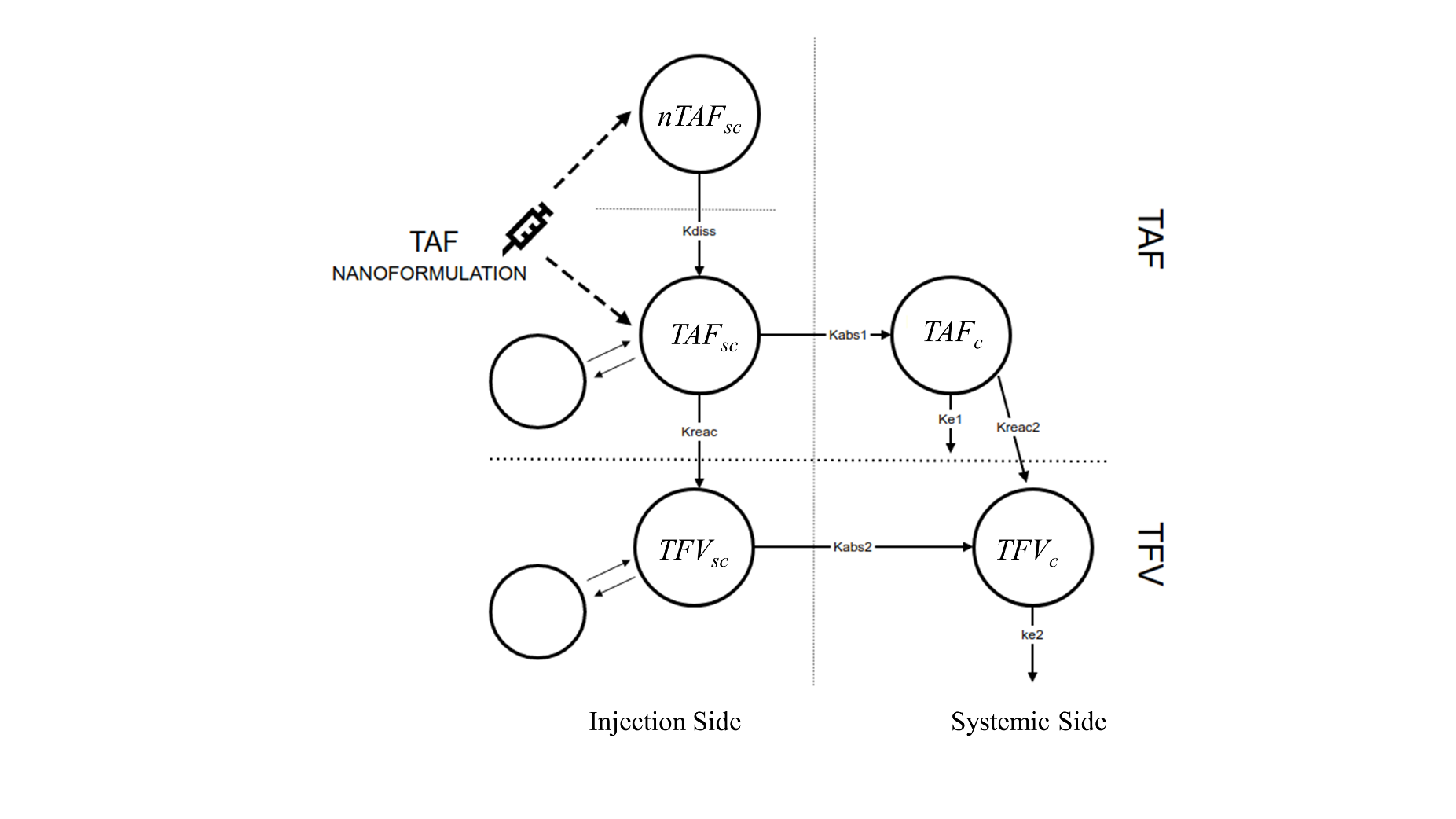


*Supplementary Figure 2.* MBPK schematics for nanoformulated TAF in the nBIC-TAF formulation. TFV sub-model included.


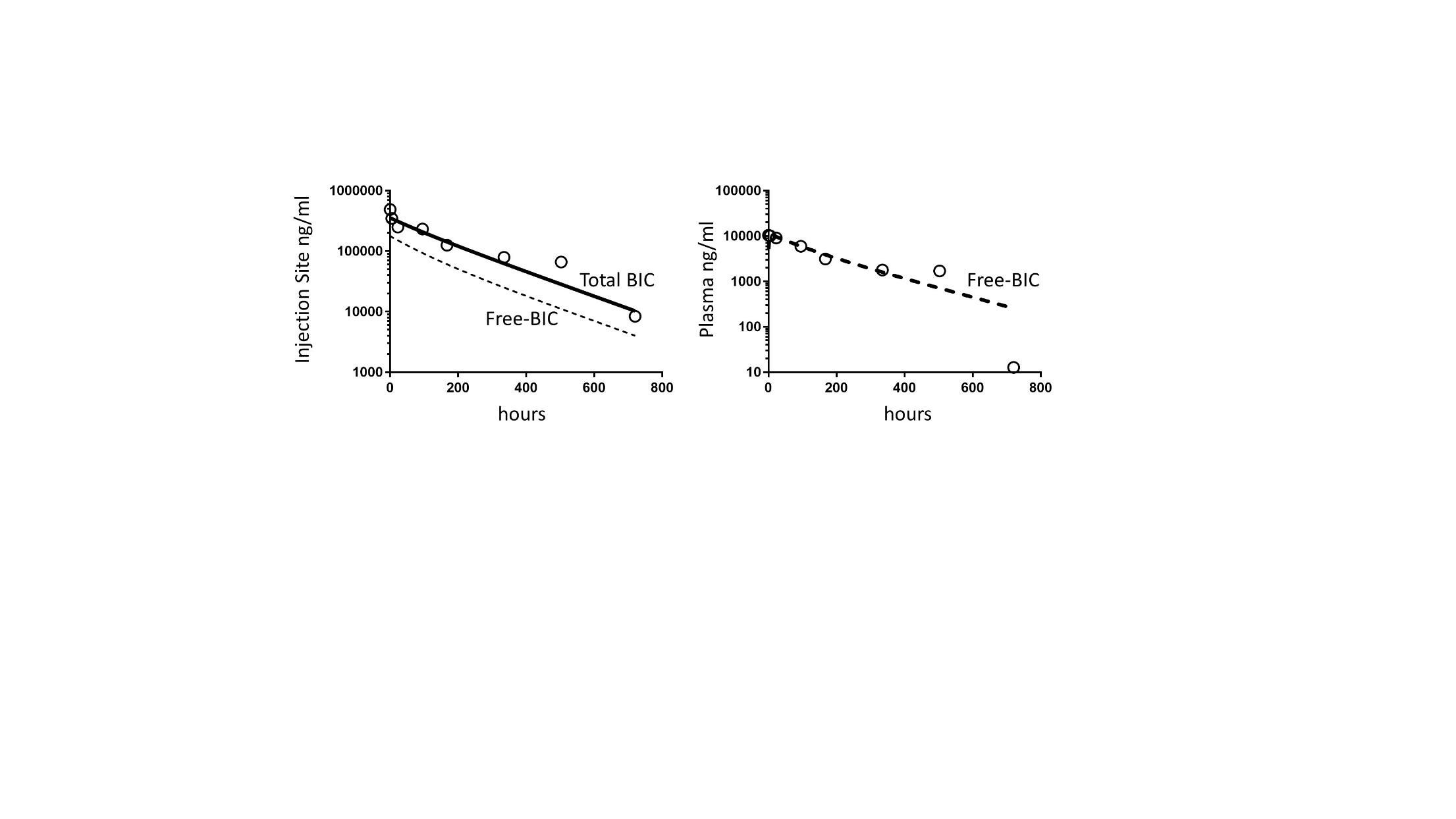


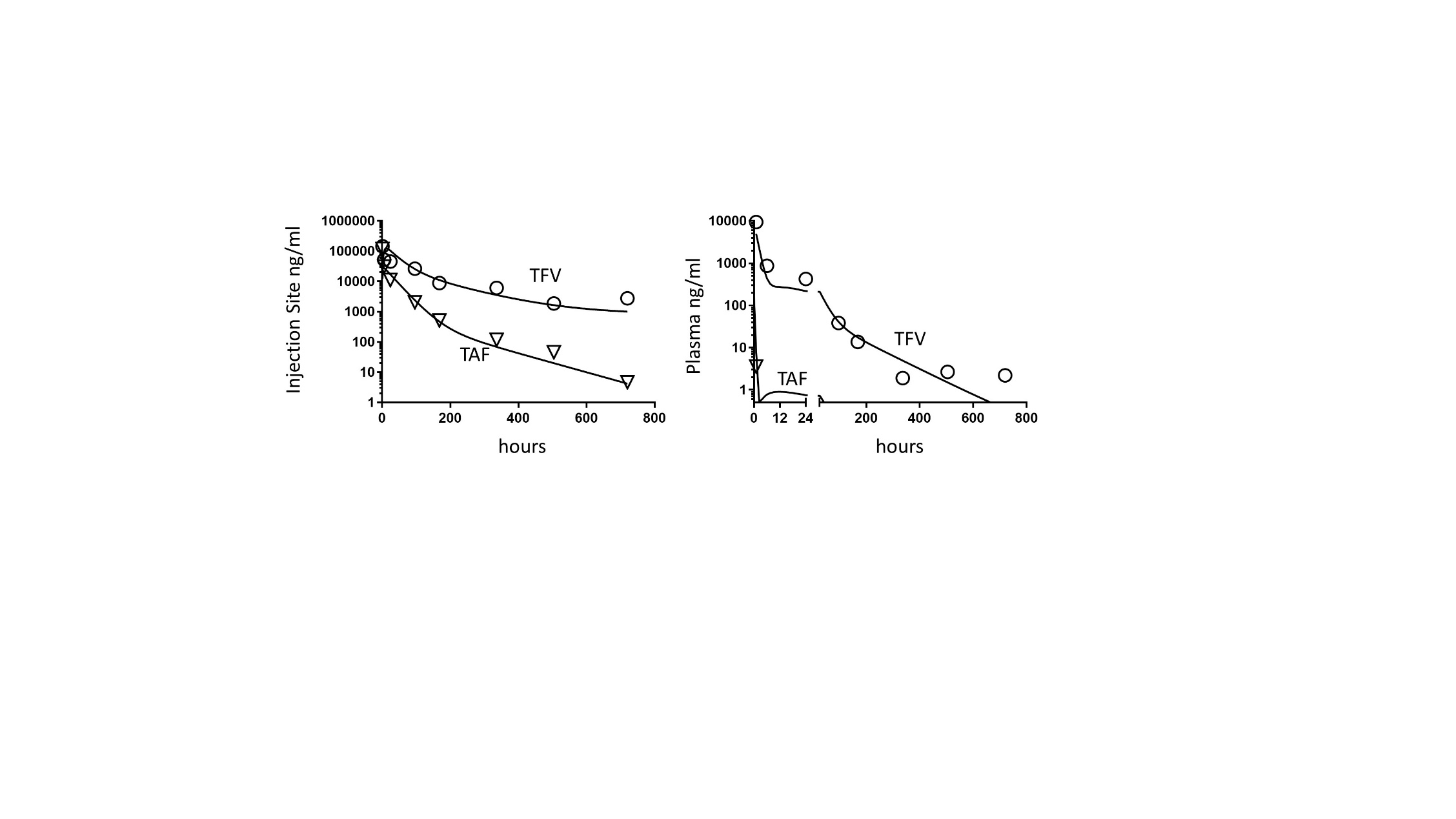


*Supplementary Figure 3.* Model fit results of the **mouse** INJECTION SITE occurring simultaneously to plasma fit (Figure 1, main text). Nanoformulated BIC (upper) and TAF-TFV (bottom).

**References**

Markowitz, M., Zolopa, A., Squires, K., Ruane, P., Coakley, D., Kearney, B., et al. (2014). Phase I/II study of the pharmacokinetics, safety and antiretroviral activity of tenofovir alafenamide, a new prodrug of the HIV reverse transcriptase inhibitor tenofovir, in HIV-infected adults. *J Antimicrob Chemother* 69, 1362–1369. doi:10.1093/jac/dkt532.

Prathipati, P. K., Mandal, S., Pon, G., Vivekanandan, R., and Destache, C. J. (2017). Pharmacokinetic and Tissue Distribution Profile of Long Acting Tenofovir Alafenamide and Elvitegravir Loaded Nanoparticles in Humanized Mice Model. *Pharm Res* 34, 2749–2755. doi:10.1007/s11095-017-2255-7.
